# Perceptual learning and neural correlates of virtual navigation in subjective cognitive decline: A pilot study

**DOI:** 10.1016/j.isci.2024.111411

**Published:** 2024-11-17

**Authors:** Amir Amedi, Shahar Shelly, Nira Saporta, Merav Catalogna

**Affiliations:** 1 The Baruch Ivcher Institute for Brain, Cognition, and Technology, Baruch Ivcher School of Psychology, Reichman University, Herzliya, Israel; 2 Department of Neurology, Rambam Medical Center, Haifa, Israel; 3 Rappaport Faculty of Medicine, Technion-Israel Institute of Technology, Haifa, Israel; 4 Remepy Health Ltd, Ramat Gan, Israel

**Keywords:** Neuroscience, Cognitive neuroscience

## Abstract

Spatial navigation deficits in age-related diseases involve brain changes affecting spatial memory and verbal cognition. Studies in blind and blindfolded individuals show that multisensory training can induce neuroplasticity through visual cortex recruitment. This proof-of-concept study introduces a digital navigation training protocol, integrating egocentric and allocentric strategies with multisensory stimulation and visual masking to enhance spatial cognition and brain connectivity in 17 individuals (mean age 57.2 years) with subjective cognitive decline. Results indicate improved spatial memory performance correlated with recruitment of the visual area 6-thalamic pathway and enhanced connectivity between memory, executive frontal areas, and default mode network (DMN) regions. Additionally, increased connectivity between allocentric and egocentric navigation areas via the retrosplenial complex (RSC) hub was observed. These findings suggest that this training has the potential to induce perceptual learning and neuroplasticity through key functional connectivity hubs, offering potential widespread cognitive benefits by enhancing critical brain network functions.

## Highlights

• Blindfolding multisensory digital training improves spatial memory performance
• Increased allocentric and egocentric navigation connectivity via the RSC hub
• Elevated MTL hub, PFC, and DMN connectivity correlated with spatial performance
• Enhanced V6, thalamus, and hippocampus network correlated with spatial performance

## Graphical abstract

**Figure:**
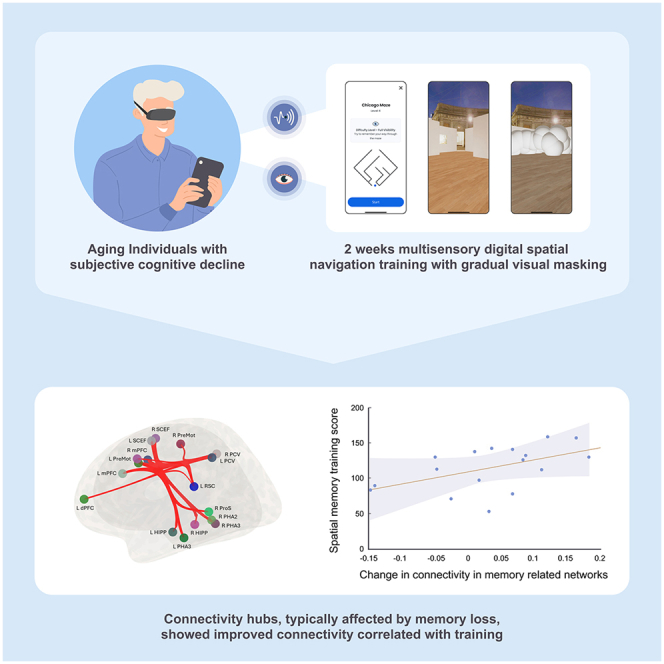

## Introduction

Spatial navigation, a fundamental aspect of human cognition, relies on the coordinated interplay of diverse multiple cognitive and multisensory processes, including visual perception, spatial orientation, learning, and memory. These intricate mechanisms work together synergistically to form spatial knowledge, enabling individuals to navigate and interact adeptly within their surroundings.^1^ Since spatial navigation involves multiple sensory cues and executive function abilities, two main strategies were suggested to form a successful navigation: the allocentric and the egocentric strategy, specified by different spatial reference frames.^2^^,^^3^ Egocentric navigation is a basic form of navigation based on self-motion and relative positioning, essential for initial exploration and path integration. This strategy encodes spatial information from the perspective of the navigator, associating specific actions with landmarks or cues encountered along the route. Conversely, allocentric navigation develops with repeated exploration, resulting in a stable, map-like knowledge of the environment. Once developed, the allocentric strategy enhances navigation efficiency by forming a cognitive map and is commonly employed in novel and unfamiliar environments (e.g., when studying a map of an unfamiliar place prior to visiting it, to inform later egocentric navigation). Both strategies are complementary and crucial for an effective navigation task.^4^^,^^5^

Individual differences in navigational ability could be influenced by several contributing factors, including innate cognitive perceptual skills, prior experiences, training, cultural influences, and neurological differences.^1^^,^^6^ Most importantly, navigational knowledge, which includes both spatial memory and spatial navigation abilities, is known to decline in both normal^7^^,^^8^^,^^9^ and pathological aging, as observed in conditions like mild cognitive decline (MCI) and dementia,^10^^,^^11^^,^^12^ Alzheimer’s disease (AD)^13^^,^^14^ and Parkinson’s disease,^15^^,^^16^ and intersubject variability typically increases with age.^17^^,^^18^ Age-related alterations in spatial navigation performance have been identified as a potential indicator, or even a biomarker, for assessing the risk of clinical progression in age-related degenerative diseases.^19^^,^^20^^,^^21^^,^^22^^,^^23^

It has been proposed that the progression of aging and pathological neurodegeneration^22^^,^^24^ unfolds with an initial impairment in grid cell firing within the entorhinal cortex,^25^ followed by cortical thinning in the precuneus and the retrosplenial complex (RSC), regions primarily associated with egocentric navigation. Subsequently, a volume decline is observed in the medial temporal lobe (MTL), including the hippocampus, and in parietal lobes, impacting both allocentric and egocentric navigation in the prodromal AD stage.^9^^,^^26^^,^^27^ Ultimately, the progression of neurodegeneration extends across the MTL and frontal lobe regions, significantly compromising the integrity of the navigation system and cognitive function.^22^

In addition to structural alternations, previous studies employing resting-state functional magnetic resonance imaging (rs-fMRI) have shown that age-related cognitive changes correlate with abnormal, degraded functional connectivity between brain regions.^28^^,^^29^^,^^30^^,^^31^^,^^32^ These changes are particularly evident in key large-scale networks, such as the default mode network (DMN) including the hippocampus, and the frontoparietal executive brain networks, affecting the coordination of brain activity.^33^^,^^34^

More specifically, previous rs-fMRI research has identified these networks functionally contributing to navigation. Key brain regions implicated in spatial navigation include the hippocampus (HP) and the entorhinal cortex within the medial temporal lobe (MTL), the parahippocampal place area (PPA), RSC, and frontal-parietal regions like the prefrontal cortex (PFC), precuneus, specifically the visual precuneus (PVC), and inferior parietal cortex.^35^^,^^36^^,^^37^^,^^38^^,^^39^ Researchers have established a complex network of interconnected brain regions within the MTL and associated areas, collectively forming the navigation network.^40^^,^^41^^,^^42^^,^^43^ An essential network closely associated with the navigation network is the default mode network (DMN),^43^^,^^44^ which is known to be particularly affected by aging and various forms of clinical brain degeneration.^45^^,^^46^^,^^47^^,^^48^ Structural and functional changes related to the hippocampus and DMN are known to reflect age-related cognitive decline and are accompanied by the accumulation of beta amyloid plaques, which are one of the hallmark pathological features of AD.^49^^,^^50^^,^^51^ Finally, the RSC^36^ emerges as a pivotal contributor to various aspects of navigational performance, both egocentric and allocentric navigation, including accurate and path environmental cues integration and participating in managing spatial knowledge.^36^^,^^52^ Understanding the connectivity alterations between the DMN, the hippocampus, and spatial navigation networks could lead to better diagnostic markers for early detection of cognitive decline.

Therapeutic settings increasingly employ enriched environments, which refer to stimulating surroundings that facilitate cognitive, physical, multisensory, and social activities, thereby enhancing overall well-being. Research in both animals and humans demonstrates that enriched multisensory environments, whether physical or psychological, induce neurogenesis and amplify brain activity, thereby improving corresponding behavioral outcomes.^53^^,^^54^^,^^55^^,^^56^^,^^57^^,^^58^^,^^59^ Notably, exposure to enriched multisensory environments has been shown to lead to significant changes in neural structure and function.^57^^,^^59^^,^^60^ Such environments contribute to improved memory and learning capabilities, the formation of new synaptic connections, enhanced neurogenesis in the hippocampus, and an increase in overall brain size.^57^^,^^58^^,^^61^^,^^62^

Concurrently, studies indicate that intentional visual deprivation, such as through blindfolding, may induce neuroplasticity, serving as a unique form of multisensory enrichment environment when combined with cross-modal stimulation.^63^ Visual processing accounts for a significant portion, around 30%–40% of the brain’s processing capacity and metabolic activity.^64^ This allocation of resources has profound implications for the structural and functional development and organization of the brain. Consequently, vision is widely regarded as the most dominant sense in humans.^65^

Studies in blind individuals indicate enhanced memory capabilities that appear to be linked to the unmasking of weak connections between visual and high-level cognitive areas, which are usually used for visual information processing in sighted individuals. Subsequent studies have also identified increased connectivity following partial visual loss.^66^^,^^67^ Research indicates that when vision is deprived in sighted individuals, the unmasking of preexisting weak connections leads to utilization of brain areas commonly correlated with vision, fully or partially supporting other functions like multisensory integration, visuo-motor skill (such as navigation and spatial cognition), and other tasks, among them high-level cognitive tasks and visual-high cognitive functions like language, memory, and reading.^68^^,^^69^^,^^70^^,^^71^^,^^72^ Notably, research from Harvard Medical School demonstrates that even short periods, for example 5 days, of blindfolding can significantly increase plasticity in the adult brain of sighted individuals.^63^ As such, visual deprivation by way of blindfolding or tasks performed through alternate sensory routes, in essence, can be seen as a method of freeing the brain to other tasks, while strengthening normally dormant, weak, or inhibited connections and networks.

Commonly used tools in studying the effects of aging on spatial perceptual learning and spatial memory are the Morris water maze and the Hebb-Williams (HW) mazes.^73^ The HW mazes, known for their more intricate and challenging design, offer a comprehensive assessment environment and are in use in both animal models and humans.^74^^,^^75^ Our recent study demonstrated that congenitally blind individuals can develop selective activation in the visual Area V6, part of the dorsal stream involved in spatial navigation, after a brief period of cognitive training using a sensory substitution device (SSD) that conveys spatial visual information of HW mazes into sounds.^76^ Similarly, sighted individuals, when blindfolded and trained on HW mazes, showed comparable activation in Area V6.^77^ This area, crucial for egocentric navigation, responds to dynamic and static visual cues relevant to navigation^78^^,^^79^^,^^80^^,^^81^^,^^82^ and is linked to the PPA, indicating that it plays a role in spatial navigation.^83^ These findings suggest potential training methods to enhance spatial cognitive abilities in individuals at risk for AD, such as older adults or those with mild cognitive impairment.^76^^,^^84^^,^^85^^,^^86^^,^^87^^,^^88^ However, the practical challenges of implementing such training on a large scale include its time-consuming nature and the need for medical oversight. Therefore, we propose investigating whether shorter blindfolding periods, for a few hours per week, utilizing digital interventions, can produce comparable effects.

In the current proof-of-concept study, we explored a flexible digital environment designed to facilitate spatial navigation and memory tasks integrating both allocentric and egocentric strategies. This approach included audiovisual elements and gradual vision masking to increase task difficulty and induce neuroplasticity.

We proposed two hypotheses to examine the effect of this approach: (1) our digital unsupervised multisensory training protocol may be an effective tool to induce perceptual learning based on partial vision and auditory cues. (2) Our proposed intervention could potentially stimulate neuroplasticity through pivotal functional connectivity hubs, such as the MTL and RSC. These regions are particularly vulnerable to age-related changes and are known to exhibit widespread connectivity across the brain, suggesting that improvements in their function may have cascading effects throughout the brain.

To test our hypothesis, we conducted a longitudinal study to assess the impact of a combined digital multisensory training on behavior and rsFC in aging individuals experiencing subjective cognitive decline (SCD). Additionally, we aimed to explore potential correlations between the observed changes in rsFC and spatial memory performance under the framework of our working hypothesis.

## Results

### Perceptual learning

**Figure 1:**
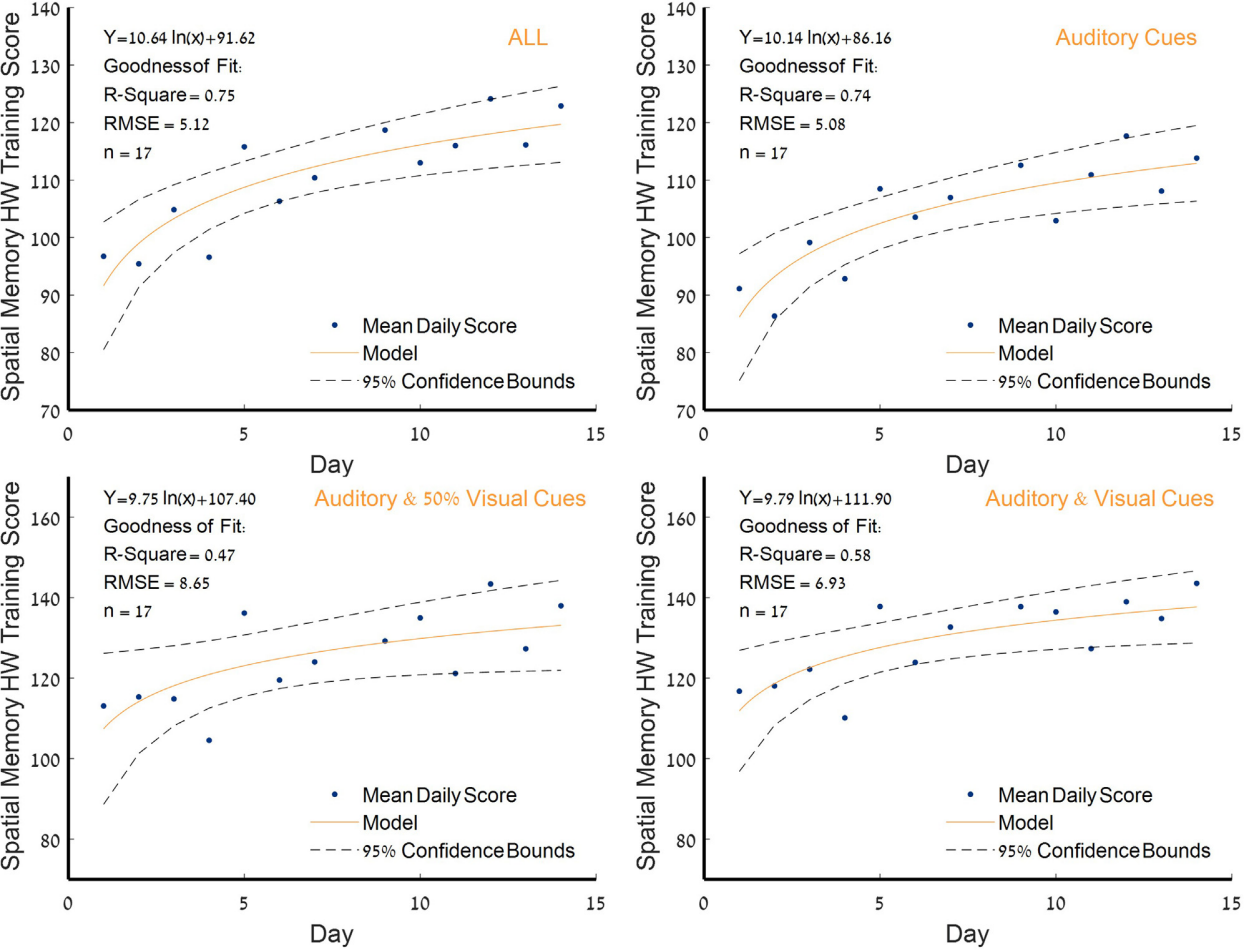
Perceptual logarithmic learning curve Best-fit analysis of the improvement in daily performance using two-parameter logarithmic model, Y = a·ln(x)+b. Data are presented with 95% confidence bounds. Spatial memory HW training score is calculated as the final daily levels divided by the daily training time. RMSE: root-mean-square error; HW, Hebb-Williams mazes.

The average daily spatial memory training score was significantly enhanced following 2 weeks of training from 96.4 ± 11.7 to 122.7 ± 13.3 with a large effect size (*p* < 0.005, d = 0.788). More importantly, perceptual learning using auditory cues (blindfolding navigation), which constitutes 63% of the total training time, was improved from 91.1 ± 8.5 to 113.8 ± 9.4, with a large effect size (*p* < 0.005, d = 0.786), whereas learning using audiovisual cues was improved from 116.7 ± 9.0 to 143.6 ± 9.6 with a medium effect size (*p* < 0.05, d = 0.652). The robust non-linear regression analysis approach was used to evaluate logarithmic model of the corresponding perceptual learning curve. The parameter estimates and 95% confidence intervals for the overall learning were as follows: a = 10.94 [CI: 6.58 14.70]; b = 91.62 [CI: 83.74 99.49]. The parameter estimates and goodness of fit statistics revealed a positive logarithmic correspondence in spatial memory HW training score through the study (Figure 1). Additional information is provided in Table S2.

### Increased anticorrelation in rsFC between V6 hub, the thalamus, and MTL

**Figure 2:**
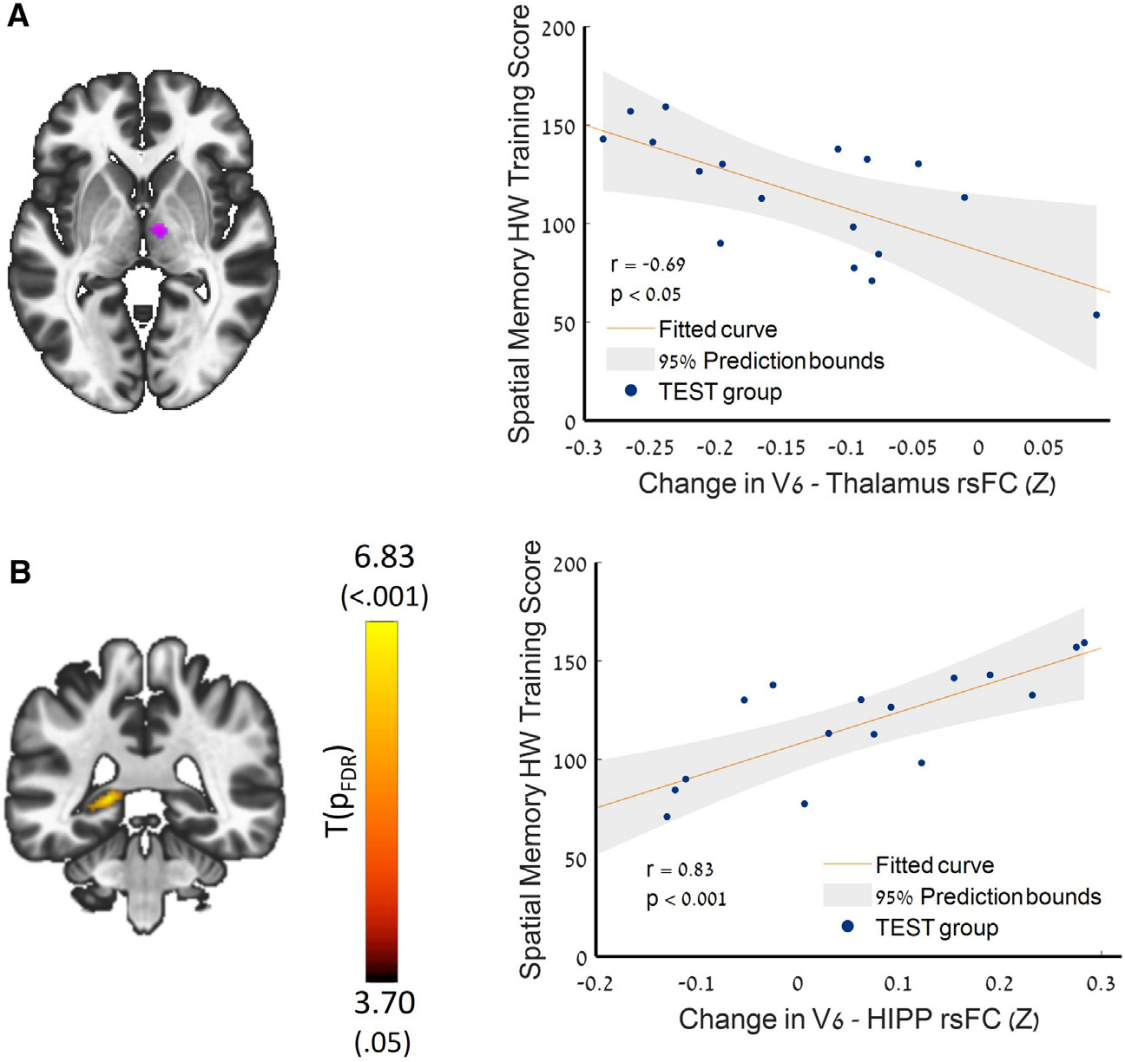
Seed-to-voxel V6 seed longitudinal differences (A) Increased post-training rsFC anticorrelation between the left visual area V6 and the right thalamus (k = 115, p_FDR_ < 0.05); a correlation plot between the changes in spatial memory HW training score and V6-thalamus post-training connectivity; r, p, spearman rank correlation coefficients. (B) Regression map and a correlation plot between changes in spatial memory HW training score and V6-hippocampus rsFC changes. Seed: left V6; *n* = 17, POST > PRE-intervention, parametric stats, two-sided. See also Table S3 for details.

Increased anticorrelation was demonstrated between the left and right visual area V6 seed and right central medial nucleus of the thalamus (k = 115, p_FDR_ = 0.049). This increase was correlated with training performance (r = −0.689, *p* < 0.05) (Figure 2A; Table S3). Significant correlation was also found between the change in left V6 and the left hippocampus rsFC with training performance (r = 0.828, *p* < 0.001) (Figure 2B).

### Increased rsFC between the MTL hub and frontal executive areas

**Figure 3:**
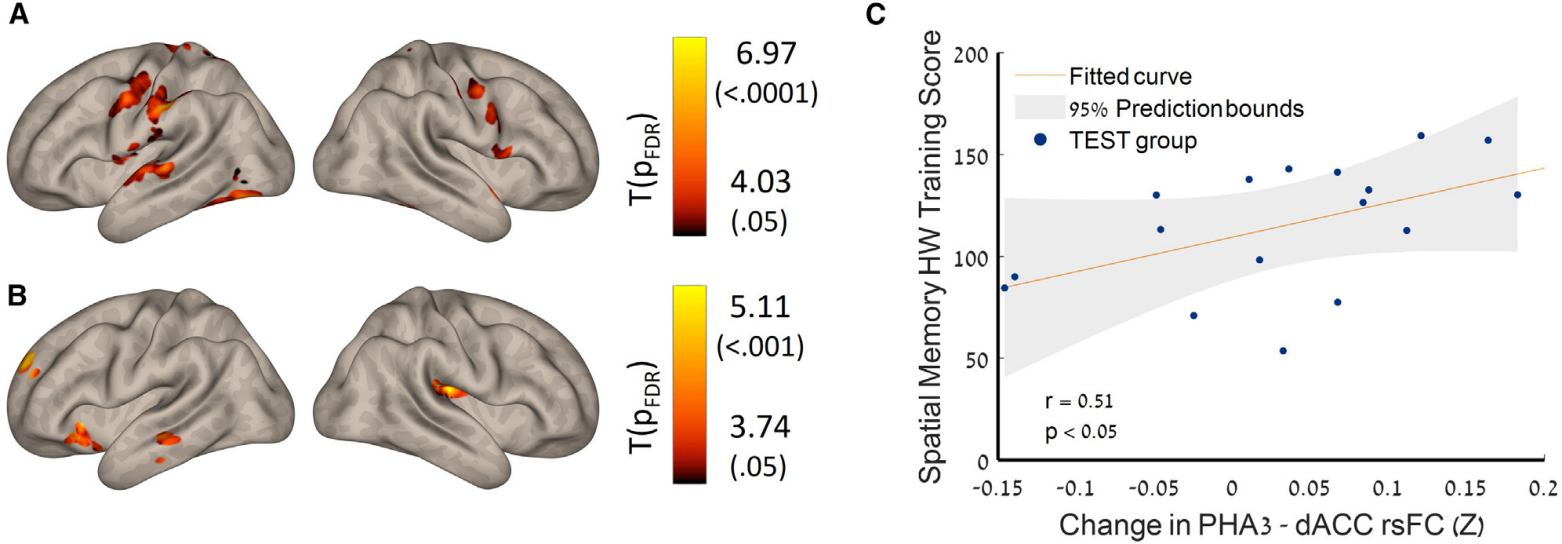
Seed connectivity maps of longitudinal differences through the MTL hub (A) Seed: left hippocampus. (B) Seed: right parahippocampal area. POST > PRE-intervention of the resting state brain imaging data—group level. *n* = 17, *p* < 0.05, FDR-corrected, parametric stats, two-sided. See also Table S3 for details. (C) Correlation between the changes in training performance and increased PHA3-dACC post-training connectivity; r, p, spearman rank correlation coefficients. PHA, parahippocampal area; dACC, dorsal anterior cingulate cortex.

Seed-to-voxel-based analysis revealed a significantly increased post-training rsFC between left and right hippocampal and PPA within the MTL and between the frontoparietal and the DMN networks (Figure 3; Table S3; Figures S3–S7).

Most significant increases in rsFC were demonstrated between the left hippocampus and the precuneus and the posterior cingulate cortex (PCC, BA31_R, k = 328, p_FDR_ < 0.0001), inferior parietal cortex, IPC (BA40, k = 305, p_FDR_ < 0.0001), and posterior parietal cortex (BA5, k = 356, p_FDR_ < 0.0001) (Figure 3A). Significant increases in rsFC were also demonstrated between the left PPA and the left dorsal prefrontal cortex (dPFC, BA9) rsFC (k = 176, p_FDR_ = 0.027) and between the right PPA and the anterior prefrontal cortex and right dorsal anterior cingulate cortex (dACC) (BA10, BA32; k = 98, p_FDR_ = 0.010, k = 158, p_FDR_ = 0.005, respectively) (Figure 3B). Importantly, this increase was correlated with training performance (r = 0.508, *p* < 0.05) and may demonstrate the dynamic learning process (Figure 3C).

### Increased rsFC of areas related to ego- and allocentric navigation

**Figure 4:**
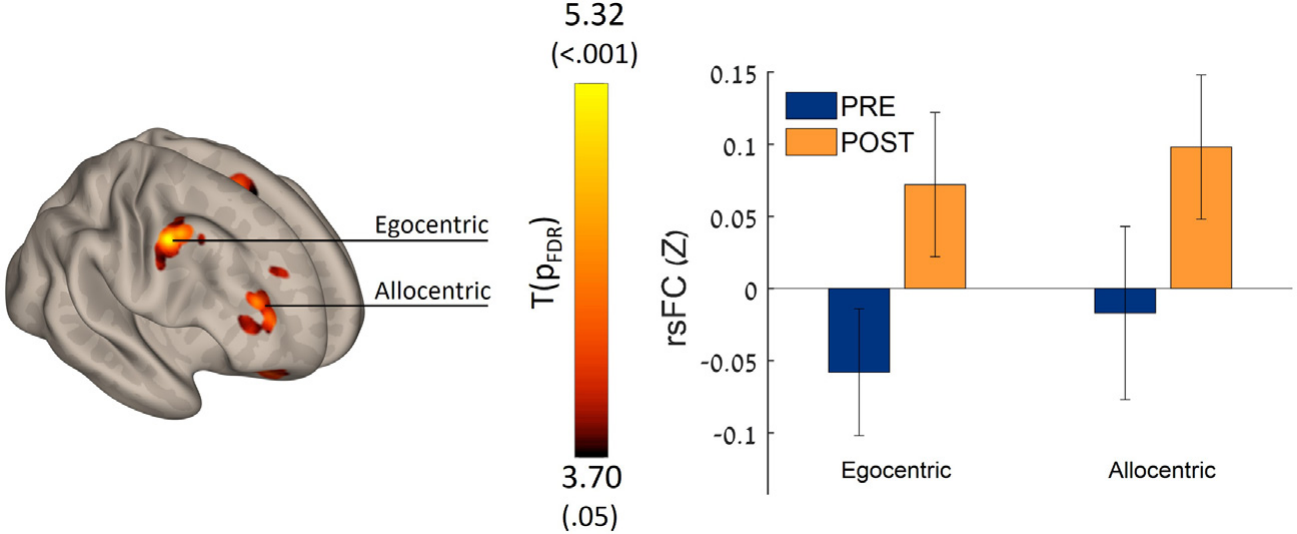
Seed-to-voxel connectivity maps of longitudinal differences through the RSC hub The retrosplenial complex (RSC) integrates both egocentric and allocentric spatial information streams. Improved post-training rsFC was found in both egocentric and allocentric networks (k = 211, p_FDR_ < 0.002 and k = 175, p_FDR_ < 0.004, respectively) Seed: right RSC, *n* = 17, POST > PRE-intervention, parametric stats, two-sided. See also Table S3 for details. Bar graphs of cluster Fisher’s Z effect size connectivity values; data are presented as mean ± CI.

As the RSC integrates both egocentric and allocentric spatial information streams, we demonstrated increased connectivity in this key area following the treatment. RsFC between the right RSC seed and right parietal cortex was increased (k = 211, p_FDR_ < 0.002), potentially contributing to the egocentric navigation performance. Additionally, increased rsFC was demonstrated in the right anterior prefrontal cortex (BA10, k = 175, p_FDR_ < 0.004), potentially contributing to the allocentric navigation performance (Figure 4; Table S3).

### Identifying a large-scale navigation network sensitive to multisensory intervention

**Figure 5:**
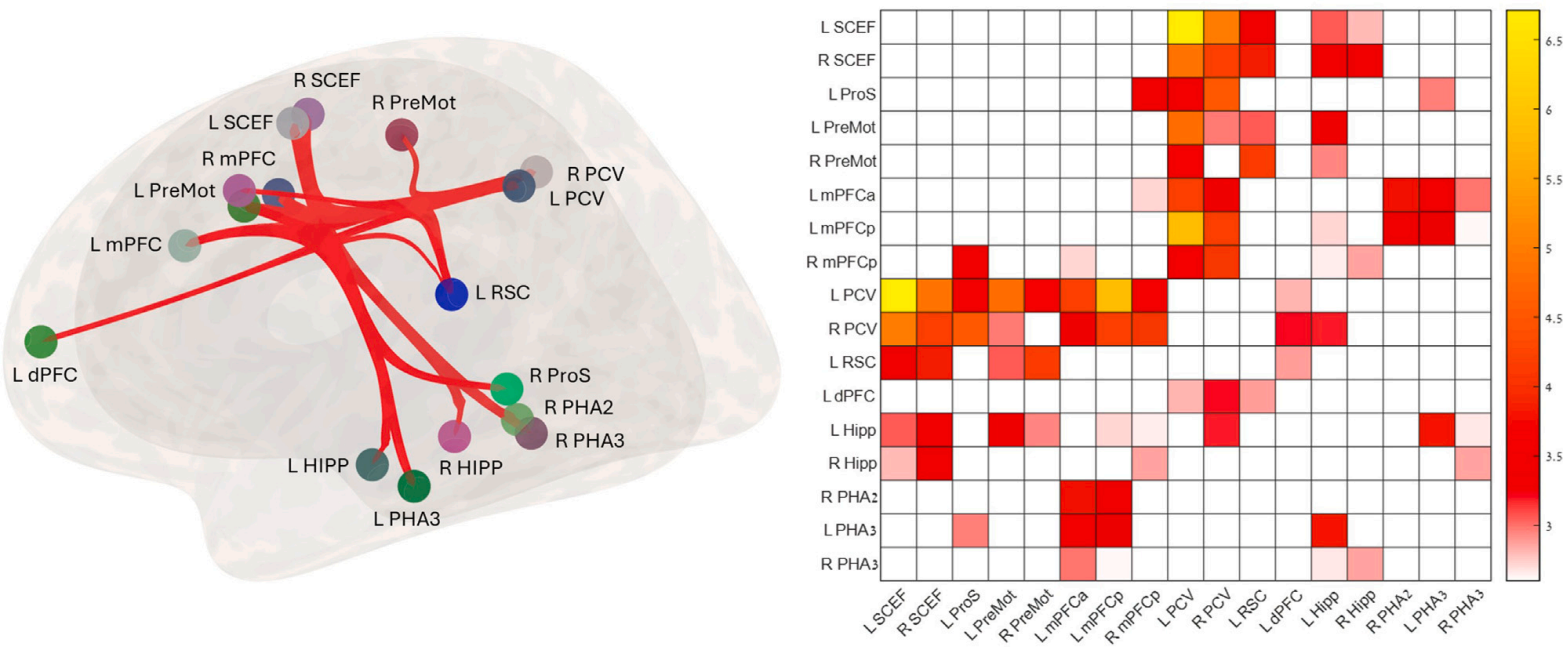
ROI-to-ROI navigation network analysis of longitudinal post-intervention differences Brain network representation and a connectivity matrix of significant post-intervention alternations. Increased rsFC was demonstrated within the frontoparietal-MTL network regions, where the retrosplenial complex (RSC) and the visual precuneus (PVC), directing and integrating the egocentric and allocentric spatial information streams. HIPP, hippocampus, PHA, para-hippocampus area, ProS, posterior cingulate, RSC, retrosplenial complex, PVC, visual precuneus, SCEF, supplementary and cingulate eye field, mPFC, medial prefrontal cortex, dPFC, dorsal prefrontal cortex, PreMot, premotor, POST > PRE-Intervention, *n* = 17, p_FDR_ < 0.05, (F POS >2.82), White = NS.

We identified 22 ROIs distributed in the MTL, and frontoparietal regions (Table S4), constructing a large-scale organization of the navigation network. Figure 5 shows an ROI-to-ROI analysis of brain network representation and a connectivity matrix of significant post-intervention alternations within these regions (p_FDR_ < 0.05). Increased rsFC was demonstrated within the frontoparietal-MTL network regions, the RSC and the visual precuneus (PVC), directing and integrating the egocentric and allocentric spatial information streams. A significant increased anticorrelation rsFC was demonstrated between the left and right visual area V6 and V6A and the central medial nucleus of the thalamus (CeM) (Figure S8).

## Discussion

In this proof-of-concept study, we explored the potential of a flexible digital environment to enhance spatial navigation and memory, focusing on large-scale modulation of synaptic connectivity and neuroplasticity in the SCD population.

This intervention uniquely incorporated both allocentric and egocentric navigation techniques through digital HW mazes and employed an innovative protocol shifting from visual to auditory cues, with gradual vision masking.

Participants engaged in a 2-week protocol of daily half-hour digital intervention and were evaluated before and after the training using rsFC. Key results showed (1) significant improvement in spatial memory performance (Figure 1); (2) increased anticorrelation between visual area V6 and the thalamus—this increase was also correlated with HW maze-training score (Figure 2); (3) an increased connectivity between the MTL, memory-related hub, and executive working memory frontal areas and DMN regions, which was also correlated with the spatial memory HW maze training score (Figure 3); and (5) a significant increase in connectivity between the allocentric and egocentric navigation areas through the RSC functional connectivity hub (Figure 4). Taken together, our results demonstrate that digital interventions can enhance rsFC within the frontoparietal-MTL network regions, including the RSC and the PVC, which direct and integrate the egocentric and allocentric spatial information streams.

Our research has yielded significant results in testing the hypotheses regarding brain plasticity induced by brief multisensory training. Firstly, our findings provide an empirical proof-of-concept for the hypothesis that short exposure to digital multisensory navigational strategy protocol effectively promotes neuroplasticity in a very specific and predictive way.

In line with our hypothesis proposing pivotal functional connectivity hubs effect, the proposed multisensory training appears to be a promising approach for enhancing brain plasticity through certain hubs (MTL, RSC, and V6). Importantly, the observed alterations in brain connectivity primarily implicated navigation networks, known to be associated with learning, retrieval, coordination, and control, as evidenced by prominent changes in frontal brain regions. Notably, the specificity of these changes to navigation, particularly the interplay between allocentric and egocentric processing mediated by the RSC, underscores its pivotal role in integrating diverse navigation information streams.^36^ Moreover, the correlation between changes in brain connectivity and navigation capability further supports the strong perceptual learning effect.

If future studies are able to generalize the results of this proof-of-concept study to larger, more diverse populations, in controlled trials and over a longer period of time, this approach has the not only potential to delay neurodegenerative processes within the aging brain but also to mitigate them. By specifically targeting and modulating brain regions vulnerable to aging, characterized by widespread connectivity across the brain, enhancing in their function may have cascading effects throughout the brain.

### Providing a multisensory enriched environment through perceptual learning protocols

In this study, we explored the ability of learning of spatial information acquired through both visual and non-visual cues in a virtual environment, by conveying information typically received through vision and transferring it to a different sense—audio. Our results demonstrate a logarithmic learning curve for both visual and perceptual audio training following a 2-week period of self-training unsupervised protocol.

Experimental research exploring tailored-made technologies with perceptual learning protocols has revealed the brain’s ability for neuroplasticity and reconstruction throughout life.^63^^,^^71^^,^^89^^,^^90^^,^^91^^,^^92^^,^^93^ Additionally, much of our understanding of these underlying mechanisms comes from studies involving individuals deprived of a sense, such as congenitally blind or late blind individuals.^76^^,^^90^^,^^93^^,^^94^^,^^95^^,^^96^^,^^97^ Specifically, research employing visual to auditory sensory-based training has shown that extensive training leads to activation of the occipital cortex.^92^^,^^97^^,^^98^^,^^99^^,^^100^

Visual area V6, inherently a retinotopic area in nature, but also constitutes a motion-sensitive region that is specialized in processing visual stimuli from the peripheral areas of the visual field, particularly responsive to translational motion.^78^^,^^80^ In our previous functional activity studies, we demonstrated that area V6 is selectively involved in multisensory egocentric navigation independently of the sensory modality used in blind and sighted population, suggesting that area V6 is a unique hub that transforms spatially relevant sensory information into an egocentric representation for navigation.^76^ Additionally, area V6 can be recruited for spatial computations independently of visual modality or even visual experience.^77^ In the current study, we identified increased anticorrelation between left and right V6 and the central medial thalamus (CMT). The thalamus serves as an integrative hub for transmitting sensory information. Specifically, the CMT has been proposed as a central hub for merging affective, cognitive, and sensorimotor functions essential for guiding goal-directed behaviors, through the striatal, limbic, and motor systems within the forebrain.^101^^,^^102^ It has been shown that rsFC between the visual area and the thalamus is anticorrelated in healthy subjects.^103^ Taken together, our results may reflect recruitment of V6-thalamic pathway, suggesting improved sensory transmission involved in egocentric navigation. Additionally, we demonstrated a significant correlation between increased V6-MTL rsFC and HW training score. This result strengthens the role of area V6, which is connected with the PPA, as a network hub that may have a role also in allocentric navigation pathways.^83^^,^^104^ Based on our findings, we propose that interventions utilizing sensory substitution could create powerful digitally enhanced cross-modal environments.

### Normalizing brain connectivity patterns in aging individuals with SCD

Spatial memory is one of the first cognitive functions that deteriorates with aging, especially in pathological cases such as dementia and AD.^8^^,^^105^^,^^106^ Numerous studies have shown that both subjective and objective decline in spatial abilities occur with aging. The exact nature of the breakdown in these networks' connectivity is still being debated, with some studies showing dysfunction in either egocentric or allocentric functions or switching between the two of them.^22^^,^^107^ However, the interaction between these networks undergoes significant age-related alterations that correlate with the decline in function. This decline is linked to abnormal interactions between the egocentric and allocentric navigation networks, and it is also thought to be tied to the deterioration of memory and executive functions. The present study’s results suggest a significant enhancement in the connectivity of crucial networks following training, particularly through two main connectivity hubs: the RSC and the MTL, including the precuneus, hippocampus, and the PPA and certain connection to the DMN.

Our results show increased connectivity between the RSC connectivity hub and certain regions of the egocentric and allocentric navigation networks. This observation is in line with previous study in SCD subjects who demonstrated decreased connectivity vs. healthy controls.^36^ Alternation in the RSC rsFC network was also demonstrated in MCI and AD.^14^^,^^22^^,^^108^^,^^109^ Our findings indicate that training in an integrative navigation strategy may enhance visuospatial learning skills and the ability to shift between egocentric and allocentric representations by promoting increased connectivity of the RSC, a pivotal hub for translating and interacting information between these two cognitive frameworks.

The DMN is known to be functionally connected to the hippocampus,^110^ critical for both spatial memory, especially allocentric-map-based navigation, and verbal memory, as well as other intrinsic functions.^40^^,^^41^^,^^42^ The hippocampus plays a crucial role in creating cognitive maps, which enable organisms to navigate through their environments effectively by encoding spatial information and integrating sensory and self-motion cues.^111^ Aging and clinical brain degeneration particularly affect these regions.^112^^,^^113^^,^^114^ Our findings indicate that enhanced connectivity between the MTL hub, particularly the hippocampus and PPA, along with frontoparietal regions, potentially facilitates the restoration of allocentric navigation pathways. Notably, this increased connectivity exhibited a positive correlation with improved learning performance among participants. The hippocampal-parietal network is thought to play a role in the organization and retrieval of externally oriented spatial knowledge^115^; therefore, our results imply that increased connectivity might serve as a compensatory mechanism for deficits in spatial abilities.

In conclusion, our findings suggest that digital training approach integrating egocentric and allocentric strategies, and audio-vision multisensory techniques, may enhance spatial cognitive performance and neuroplasticity via key functional connectivity hubs such as MTL, RSC, and V6. These regions, prone to age-related changes, possess extensive connectivity across the brain, implying that improvements in their function may yield widespread cognitive benefits. Additionally, by challenging the critical periods theory for brain plasticity in aging, our findings suggest that relatively brief daily digital interventions could induce significant alterations in brain connectivity within regions crucial for spatial and verbal memory, areas often affected in early stages of AD.

### Limitations of the study

This study has several limitations. Firstly, the homogeneity in clinical characteristics within our participant group due to a narrow age range, and a relatively small sample size, might restrict the generalizability of our findings to the broader population. However, this is quite reasonable and commonplace in the first proof-of-concept study that was primarily designed as a neuroimaging study aiming to identify a large-scale navigation network sensitive to multisensory intervention. Future research should replicate this study in a larger sample size to allow examination of subgroup differences in personal learning strategies and effect of baseline cognitive and emotional state on neuroplasticity and navigation performance. Secondly, although our study recruited healthy aging participants, we did not conduct a comprehensive neuropsychological assessment specifically targeting visuo-spatial skills. As a result, we might have missed identifying individuals with exceptional or notably low spatial memory abilities. Additionally, data were gathered following a 2-week intervention period. Assessing the prolonged impact remains to be determined. Lastly, although the correlation between the changes in connectivity patterns and the training score suggests causality, incorporating a control group, trained with other established digital cognitive training methods in future studies, would significantly enhance the robustness of the findings. Our follow-up research is also designed to investigate additional neuroplasticity mechanisms such as diffusion tensor imaging (DTI) microstructure changes and morphometry, specifically in populations diagnosed with neurodegenerative diseases such as MCI and early AD.

## Resource availability

### Lead contact

Further information and requests for resources should be directed to and will be fulfilled by the lead contact, Prof. Amir Amedi (amir.amedi@runi.ac.il).

### Materials availability

This study did not generate new unique materials.

### Data and code availability

• Data and code reported in this article will be shared by the lead contact upon request.
• All original code reported in this article will be shared by the lead contact upon request.
• Any additional information required to reanalyze the data reported in this article is available from the lead contact upon request.

## Acknowledgments

We would like to thank Dr. Ofer Tur-Sinai for commenting on an earlier draft of this paper. We would like to thank Yoad Ben-Adiva for managing the trial and Rotem Vekslar, Ya’ira Somerville, and Sofia Sacal from the Institute for Brain Cognition and Technology, as well as Maya Goldberg and Gal Yogev from Remepy, for study coordination and data collection. We wish to thank Dr. Amber Maimon for helping with the literature review and with commenting on an earlier version of the introduction and discussion of the paper. Our appreciation also extends to Lior Benderski, Johnathan Amit Kanarek, and Shahar Har Nesher from Remepy for their assistance in programming and designing the app and to Ariel Shahaf for his contributions to the development of psychological intervention protocols. We wish to thank Dr. Shai Erlich for his advice regarding the protocol. Special recognition is due to Dr. Dikla Ender-Fox and Dalit Shlayn from the Ruth and Meir Rosenthal Brain Imaging Center at Reichman University for their support with brain scanning. Finally, we would like to express our deepest gratitude to Dr. Michal Tsur and Or Shoval from Remepy for their visionary leadership and profound insights on the project and their help in executing and coordinating this complex project. This research was supported by the European Union's Horizon 2020 research and innovation program under grant agreement No 101017884 and the European Research Council under the European Union's Horizon 2020 research and innovation program grant agreement No 773121.

## Author contributions

A.A. and M.C. conceived the study; M.C. formally analyzed the data; M.C. and A.A. investigated and interpreted the data; N.S. designed the clinical intervention; M.C., A.A., S.S., and N.S. wrote and revised the manuscript; A.A. contributed to the founding acquisition.

## Declaration of interests

A.A. is a co-founder and employee of Remepy Health Ltd. M.C. and N.S. are employees of Remepy Health Ltd. and they hold stock options of Remepy, Inc., S.S. is a consultant of Remepy Health Ltd., and he holds stock options of Remepy, Inc. A.A., S.S., and N.S. are named inventors in patent applications filed by Remepy Health Ltd. in connection with the App.

## STAR★Methods

### Key resources table

**Table:** 

REAGENT or RESOURCE	SOURCE	IDENTIFIER
**Software and algorithms**		

MATLAB	R2021b	https://www.mathworks.com
CONN	v22a	https://web.conn-toolbox.org
SPM12	SPM12	http://www.fil.ion.ucl.ac.uk/spm
HCP-MMP Atlas	HCPex	https://github.com/wayalan/HCPex

### Experimental model and study participant details

#### Participants

A proof-of-concept, pilot study, designed as a prospective, open-label trial, was undertaken at the Baruch Ivcher Institute for Brain, Cognition & Technology (BCT) within the School of Psychology at Reichman University (RUNI), Israel. In this study we recruited twenty adults, males and females, aged between 55 and 60 who exhibited signs of SCD, demonstrated a Montreal Cognitive Assessment (MoCA) score of 24 or higher.^116^ Participants were recruited through social media. Two participants were excluded due to claustrophobia, and one opted out by withdrawing consent. Consequently, the study proceeded with seventeen participants. The mean age of participants at inclusion was 57.2 ± 1.5, the mean MoCA score was 27.6 ± 1.9, and 47% were males. Subjective Cognitive Decline (SCD) characterizes individuals' self-reported experiences of increasing confusion or memory lapses, indicating a form of cognitive impairment. This condition can serve as an early sign of AD and related dementia.^117^ Exclusion criteria included: any history of malignancy, traumatic brain injury, brain surgery, chronic subdural hemorrhages, epilepsy and other neurodegenerative diseases, any psychiatric disorder or pathological cognitive decline, and MRI contraindications. The study was approved by RUNI Institutional Review Board (IRB) (No. P_2023138). The neuroimaging study protocol was reviewed and approved by the IRB of Sheba Medical Center (No. 8591-21-SMC). All participants signed an informed consent prior to their inclusion. All research was conformed to the 2013 Helsinki Declaration.

### Method details

#### Study design and digital intervention

**Figure 6:**
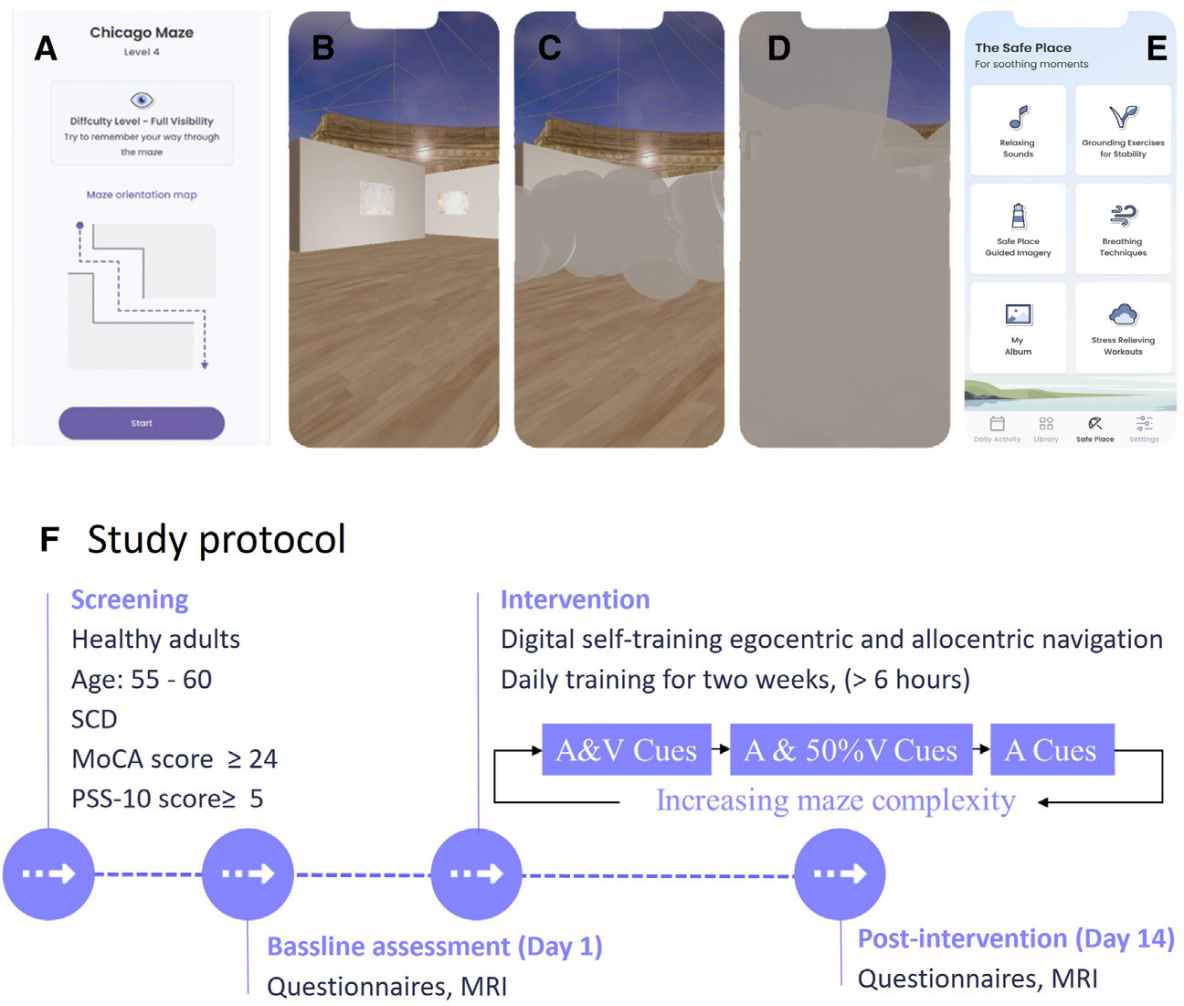
Remepy APP comprehensive training mobile application (A–F) A top view map of HW maze used. Note, the app utilizes a special version of the HW maze, which includes both an allocentric view that was introduced visually (A) and a digital 3D egocentric navigation view with visual and auditory input (B). This way subjects were encouraged to integrate both map-based allocentric navigation and egocentric navigation; 50% random screening masked—reducing half of the visual cues and encouraging subjects to integrate visual cues with the auditory cue (C); blindfolded navigation in which no visual cues were available and the subject was further instructed to use blindfolding (D); psychological interventions—video and audio aimed at chronic stress reduction, CBT, etc. (E); study protocol and timeline (F). A, auditory cue; V, visual cue.

This study utilized one group pre-post intervention design. After signing an informed consent, participants were engaged in a two-week digital intervention using a mobile application, with supervised sessions on days 1 and 14, supplemented by daily self-training at home. Participants were assessed for changes in the brain’s functional connectivity, well-being, and psychological state (Figure 6, Supplemental information 1.1). The participants utilized a comprehensive training mobile application developed by Remepy (https://www.remepy.com), which incorporates unique methodologies informed, among other things, by our prior research using HW mazes.^76^^,^^85^^,^^118^ The virtual navigation training protocol employs digitizing the entire HW mazes set (Figure S2), and audio-vision stimuli, which are designed to accelerate the learning process and improve neural activity coordination between sensory and cognitive brain networks. The unique approach embedded in the digital training app combines both egocentric and allocentric navigation strategies through a three-step blindfold training protocol, with progressively increasing navigation complexity: each new maze trial starts with a top view map of the maze, allowing the participants to employ an allocentric navigation strategy, followed by virtual 3D navigation experience, which allows the participants to also employ an egocentric navigation strategy. Initially, the navigation is completely sighted, supplemented by auditory cues. Once the participant successfully completed the fully sighted navigation, the top view of the maze map is presented again, followed by virtual 3D navigation that becomes more challenging, as 50% of the maze is randomly masked. In the final step, the top view of the maze map is no longer presented, and participants are asked to navigate the maze blindfolded, relying on spatial memory and auditory cues that help the participants assess their distance from the maze walls. (Figure 6A).

The distance audio algorithm utilizes a sound frequency conversion perceptual code where a higher frequency indicates proximity to a nearby wall, while a lower frequency signifies greater distance from the wall. Footstep sounds signal a clear passage. Participants guided their way by swiping their fingers across the touchscreen to move right or left, in 45-degree directions or to keep walking straight. While the main task was successful wayfinding, participants were instructed to find the fastest route to the exit while avoiding collisions with the walls.

To evaluate participants' perceptual learning performance, the software automatically recorded the daily number of completed mazes (trials) and the time spent on each training strategy: auditory and visual cues, auditory and 50% visual cues, and auditory cues. The auditory strategy trial was repeated three times for each maze (see Figure S2). The daily spatial memory HW training score was calculated for each training strategy by dividing the number of successfully completed trials by the total corresponding training time for that session (The daily training protocol scheme is provided in Figure S2). Success was defined as completing a trial within a time frame 3 min and achieving a trial score above 70, based on the participant’s deviation from the path and entry into an error zone (see Supplemental Information 1.2 and Figure S1 for details). Each trial was repeated until it was successful. Additionally, as part of a study of the potential to digitize interventions aimed at the emotional state of SCD patients, the application featured short stress regulation techniques based on mindfulness, attention-focusing exercises, and cognitive behavioral therapy, presented by video, audio, and interactive formats. Each daily self-training session lasted approximately 30 min, comprising about 25 min of engagement with the navigation program and 5 min dedicated to psychological interventions (see Figure 6E).

#### Brain imaging

Brain imaging MRI scans were performed on MAGNETOM Prisma 3T Scanner, configured with a 64-channel receiver head coils (Siemens Healthcare, Erlangen, Germany), at the Ruth and Meir Rosental Brain Imaging Center (MRI), Reichman University. The MRI protocol included the following sequences: Two runs of 300 volumes (9:28 min) resting state fMRI scans were acquired using a multi-band echo planar imaging sequence, CMRR EPI 2D.^119^^,^^120^ Scan parameters: TR: 1,870 ms, TE: 30 ms, flip angle: 75°, voxel size: 3.0 × 3.0 × 2.0 mm, FOV: 192, number of slices: 58 axial slices parallel to the AP-PC plane. During scanning, each participant was asked to remain still and relaxed, with their eyes fixated on a cross, and without thinking of anything deliberate. Foam pads and earplugs were employed to reduce head motion and scanning noise. Structural T1-weighted MRI scans were acquired for co-registration purposes using a T1-weighted 3D magnetization-prepared rapid gradient-echo (MPRAGE) sequence in a sagittal plane with 1 mm isotropic resolution. Sequence parameters: TR: 2,000 ms, TE: 1.9 ms, flip angle: 9°, TI: 920 ms, FOV: 256 × 256, and 176 contiguous slices. The MRI protocol also included T2-Fluid-attenuated inversion recovery (FLAIR), and susceptibility-weighted imaging (SWI) sequences, using standard parameters for clinical brain evaluation.

Additionally, psychological endpoints were collected using standard questionnaires pre and post intervention. These endpoints are to be analyzed and reported in future publications.

#### BOLD data preprocessing

Functional connectivity analysis was carried out using the CONN-fMRI toolbox v22a as implemented using statistical parametric mapping software SPM12 (http://www.fil.ion.ucl.ac.uk/spm). Functional volumes pre-processing pipeline included realignment with correction of susceptibility distortion interactions, slice timing correction, outlier detection, direct segmentation, and MNI-space normalization, with a resolution voxel size of 2.0 × 2.0 × 2.0 mm, and spatial smoothing (8 mm FWHM Gaussian kernel) steps.^121^ The preprocessing steps derived (1) the realignment covariate, containing the six rigid-body parameters characterizing the estimated subject motion, (2) the scrubbing covariate containing potential outlier scans performed with CONNs artifact detection tool (ART), and (3) the quality assurance (QA) covariate based on global signal change (>3 standard deviations from the mean image intensity) and framewise displacement (FD) scan-to-scan head-motion. Age and sex were also used as group (second level) covariates. A component-based noise correction procedure (CompCor) approach^122^ was used to identify additional confounding temporal factors controlling for physiological noise, BOLD signal present in white matter, and head motion effects. Finally, residual BOLD time series were then bandpass-filtered at a frequency range of 0.01–0.009 Hz.^121^ Individual connectivity maps were generated using the seed-to-voxel approach. We examined rsFC using *a priori* seeds derived from the extended HCP-MMP atlas (HCPex),^123^ a modified and extended version of the Human Connectome Project-MultiModal Parcellation atlas (HCP-MMP),^124^ which provides the surface-based of 360 human cortical areas. Bivariate correlation analysis was used to determine the linear association of the BOLD time series between the seed and significant voxel clusters. Fisher’s Z transformation was applied to the correlation coefficients to satisfy normality assumptions. Then, functional connectivity maps were thresholded at *p* < 0.05 false discovery rate (FDR) corrected for multiple comparisons.^121^ Finally, participants with head motions of >2 mm in any direction between volumes, rotations of >2° in any axis during scanning, or mean FD of >0.5 in either the pre- or post-treatment maps were excluded from the dataset.

### Quantification and statistical analysis

#### Descriptive statistics

The demographics and continuous data are expressed as means ± standard deviations (SD). Categorical data were expressed in numbers and percentages. To evaluate the intervention’s effect, the Student’s t test was used to compare post-treatment and pre-treatment data. Non-linear robust regression analysis was performed using the Nonlinear Least Squares (NLS) method. The model parameters estimates were iteratively determined using the Levenberg-Marquardt optimization method. A two-parameter logarithmic model, Y = a·ln(x)+b, was evaluated. Effect size was evaluated using Cohen’s d method. Data analysis was performed using MATLAB R2021b (MathWorks, Natick, MA) Statistics and Machine Learning toolbox, and Curve Fitting Toolbox.

#### Imaging analysis statistics

At the group level, seed-to-voxel rsFC was analyzed using a repeated measure model to test the intervention’s effect. The analysis was implemented in SPM software (version 12, UCL, London, UK) with a parametric analysis approach across the entire brain volume.^121^^,^^125^ RsFC was considered significant at joint-probability thresholds of 0.001 at the voxel level, and *p* < 0.05 false discovery rate using the Benjamini-Hochberg FDR procedure^126^ corrected for multiple comparisons across the whole brain at the cluster level, with a minimum cluster size of 50 voxels. The REX toolbox was used to extract cluster connectivity statistical values.^121^ Next, ROI-to-ROI analysis was performed to identify relationships between brain regions sensitive to the intervention. The analysis nodes were chosen based on our brain-level seed-voxel analysis results, aligned with the HCP-MMP atlas parcellation, and also based on their inclusion in a recent meta-analysis, defining a large-scale comprehensive organization of the navigation network.^127^ A bivariate group level regression analysis with behavioral covariate model was used to identify global brain correlations. Spearman rank correlations were used to test associations with perceptual learning and training scores.
