# Supplementary material for: Perceptual learning and neural correlates of virtual navigation in subjective cognitive decline: A pilot study
Source: iScience. 2024 Nov 17;27(12):111411. doi: 10.1016/j.isci.2024.111411 (PMC11634985; doi:10.1016/j.isci.2024.111411)
Supplement: Document S1. Figures S1–S8 and Tables S1–S4 [file mmc1.pdf]

iScience, Volume *27*

Supplemental information

Perceptual learning and neural correlates of virtual navigation in subjective cognitive decline: A pilot study

Amir Amedi, Shahar Shelly, Nira Saporta, and Merav Catalogna

Perceptual learning and neural correlates of virtual navigation in subjective cognitive decline: A pilot study

Amir Amedi[1,5*], Shahar Shelly[2,3], Nira Saporta[4], Merav Catalogna[1*]

[1]The Baruch Ivcher Institute for Brain, Cognition, and Technology, Baruch Ivcher School of Psychology, Reichman University, Herzliya, Israel, [2]Department of Neurology, Rambam Medical Center, Haifa, Israel, [3]Rappaport Faculty of Medicine, Technion-Israel Institute of Technology, Haifa, Israel, [4]Remepy Health Ltd, Ramat Gan, Israel, [5]Lead contact

*Correspondence: amir.amedi@runi.ac.il (A.A.), merav@catalogna.co (M.C.)

Supplemental information

Table of contents

1 Supplemental Tables 2

Table S1: Baseline characteristics 2

Table S2: Spatial Memory performance 2

Table S3: Seed-to-voxel based rsFC analysis 3

Table S4: ROI-ROI analysis nodes 3

2 Supplemental Figures 5

Fig S1. Maze setup and error calculation example. 5

Fig S2. Maze training daily protocol scheme. 5

Fig S3. Seed-to-voxel R hippocampus connectivity maps of longitudinal differences 6

Fig S4. Seed-to-voxel L hippocampus connectivity maps of longitudinal differences 6

Fig S5. Seed-to-voxel R parahippocampus connectivity maps of longitudinal differences. 7

Fig S6. Seed-to-voxel L parahippocampus connectivity maps of longitudinal differences. 7

Fig S7. Seed-to-voxel R parahippocampus connectivity maps of longitudinal differences 8

Fig S8. ROI-to-ROI navigation network analysis of longitudinal post-intervention differences. 8

3 References 9

1 Supplemental Tables

Table S1: Baseline characteristics

N	17
Age	57.2±1.5
Gender (M)	8 (47.1)
Years of education	16.1±1.7
**Marital status**	
Married/Live with a partner	13 (76.5)
Divorced	4 (23.5)
Number of children	3.1±0.9
PSS-10 (Perceived Stress Scale)	15.1±4.5
MoCA (Montreal Cognitive Assessment)	27.6±1.9
BMI (Kg/m2)	25.7±2.5

Mean ± SD (%)

Table S2: Spatial Memory performance

Cues	Time %	**DAY1**	**DAY14**	**p-value**	**a***	**a[CI]**	**b***	**b[CI]**
Auditory - Visual	18	116.7±9.0	143.6±9.6	< 0.05	9.79	[14.34 15.33]	111.9	[101.24 122.49]
Auditory - 50%Visual	19	113.1±5.8	138.0±10.1	< 0.05	9.79	[2.88 16.61]	107.4	[94.12 120.7]
Auditory	63	91.1±8.5	113.8±9.4	< 0.005	10.14	[6.10 14.17]	86.16	[78.35 93.98]
All	100	96.4 ± 11.7	122.7 ± 13.3	< 0.005	10.94	[6.58 14.70]	91.62	[83.74 99.49]

* Parameter estimates of fit logarithmic statistics

Table S3: Seed-to-voxel based rsFC analysis.

Peak changes in post-treatment functional connectivity

Seed-HCPex index*	Seed-HCPex area*	R/L	Cluster (x,y,z)	size	p-FWE	p-FDR	p-unc	BA
80	HIPP	L	-30 -60 -02	895	0.000	0.000	0.000	19
80	HIPP	L	-10 -38 +68	356	0.000	0.000	0.000	5
80	HIPP	L	-56 -06 +42	349	0.000	0.000	0.000	4
80	HIPP	R	+12 -50 +48	328	0.000	0.000	0.000	31
80	HIPP	L	-58 -20 +34	305	0.000	0.000	0.000	40
80	HIPP	L	-66 -26 +04	290	0.000	0.000	0.000	22
80	HIPP	L	-02 -20 +56	248	0.000	0.000	0.000	6
80	HIPP	R	+56 +14 -08	119	0.008	0.002	0.000	38
80	HIPP	R	+54 +06 +34	102	0.018	0.004	0.000	6
80	HIPP	L	-02 -02 +42	97	0.024	0.005	0.001	24
80	HIPP	R	+52 -06 +46	87	0.040	0.007	0.001	6
80	HIPP	L	-32 -04 +10	86	0.042	0.007	0.001	13
80	HIPP	L	-20 -56 +60	64	0.135	0.022	0.004	7
80	HIPP	R	+42 -44 -26	62	0.151	0.023	0.004	37
260	HIPP	R	+48 -76 -06	208	0.001	0.002	0.000	19
260	HIPP	L	-54 -24 +36	196	0.002	0.002	0.000	40
260	HIPP	R	+40 +16 +02	184	0.003	0.002	0.000	13
260	HIPP	L	-40 -42 -22	175	0.004	0.002	0.000	7
260	HIPP	L	-26 -82 -12	125	0.023	0.009	0.001	18
260	HIPP	R	+34 +00 -02	115	0.033	0.011	0.001	Putamen
260	HIPP	R	+46 +10 +34	108	0.044	0.012	0.001	44
87	PHA3	R	+08 +40 +32	176	0.033	0.027	0.001	9
87	PHA3	L	-08 +12 +42	140	0.089	0.050	0.002	32
266	PHA2	R	+40 +16 +02	195	0.019	0.023	0.000	13
266	PHA2	L	-12 +22 +34	163	0.046	0.028	0.001	32
266	PHA2	L	-34 +18 -02	135	0.099	0.042	0.002	13
267	PHA3	L	-04 +44 +18	158	0.006	0.005	0.000	32
267	PHA3	L	-28 +12 -12	123	0.021	0.009	0.001	Insula
267	PHA3	L	-54 -24 -14	98	0.057	0.010	0.002	21
267	PHA3	L	-16 +52 +24	97	0.059	0.010	0.002	10
267	PHA3	R	+34 -22 +16	97	0.059	0.010	0.002	Insula
132	RSC	R	+50 +06 +48	211	0.001	0.002	0.000	6

132	RSC	R	+42 +54 +12	175	0.004	0.004	0.000	10
132	RSC	R	+24 +44 -16	133	0.016	0.010	0.001	47
132	RSC	L	-28 -80 -18	110	0.039	0.021	0.001	34
132	RSC	L	-08 +06 +58	104	0.050	0.023	0.002	6
8	V6	R	+06 -08 +02	115	0.150	0.050	0.003	Thalamus
188	V6	R	+04 -18 -03	437	0.004	0.001	0.000	Thalamus

* **Glasser** atlas [1], L, left, R, right, FWE, familywise error, FDR, false discovery rate, X, Y, Z MNI coordinates, BA, Brodmann area

Table S4: ROI-ROI analysis nodes

Brain region	HCPex index*	Cluster (x,y,z)		
Visual Area 6	HCPex_2mm.8 L V6 8	-15	-79	31
Visual Area 6A	HCPex_2mm.9 L V6A 9	-21	-86	42
Supplementary and Cingulate Eye Field	HCPex_2mm.40 L SCEF 40	-5	5	59
PreMotor Area	HCPex_2mm.41 L 55b 41	-47	-1	50
Hippocampus	HCPex_2mm.80 L Hipp 80	-25	-24	-15
ParaHippocampal Area 3	HCPex_2mm.87 L PHA3 87	-31	-37	-18
PreCuneus Visual Area	HCPex_2mm.128 L PCV 128	-6	-51	48
Posterior Cingulate Cortex	HCPex_2mm.131 L ProS 131	-20	-55	4
RetroSplenial Complex	HCPex_2mm.132 L RSC 132	-4	-34	22
Anterior Cingulate Cortex	HCPex_2mm.142 L a32pr 142	-7	31	29
Anterior Cingulate Cortex	HCPex_2mm.147 L p32pr 147	-7	16	39
Dorsolateral Prefrontal Cortex	HCPex_2mm.176 L a9-46v 176	-40	52	11
Visual Area 6	HCPex_2mm.188 R V6 188	19	-77	31
Visual Area 6A	HCPex_2mm.189 R V6A 189	22	-83	45
Supplementary and Cingulate Eye Field	HCPex_2mm.220 R SCEF 220	7	6	59
PreMotor Area	HCPex_2mm.221 R 55b 221	51	2	48
Hippocampus	HCPex_2mm.260 R Hipp 260	42	-1	-1
ParaHippocampal Area 2	HCPex_2mm.266 R PHA2 266	33	-34	-12
ParaHippocampal Area 3	HCPex_2mm.267 R PHA3 267	33	-37	-15
PreCuneus Visual Area	HCPex_2mm.308 R PCV 308	7	-50	49
Anterior Cingulate Cortex	HCPex_2mm.327 R p32pr 327	11	15	39
Central Medial Thalamus	HCPex_2mm.362 L CeM 362	-3	-9	0
Central Medial Thalamus	HCPex_2mm.395 R CeM 395	6	-8	0

* **Glasser** atlas [7], L, left, R, right, X, Y, Z MNI coordinates

2 Supplemental Figures

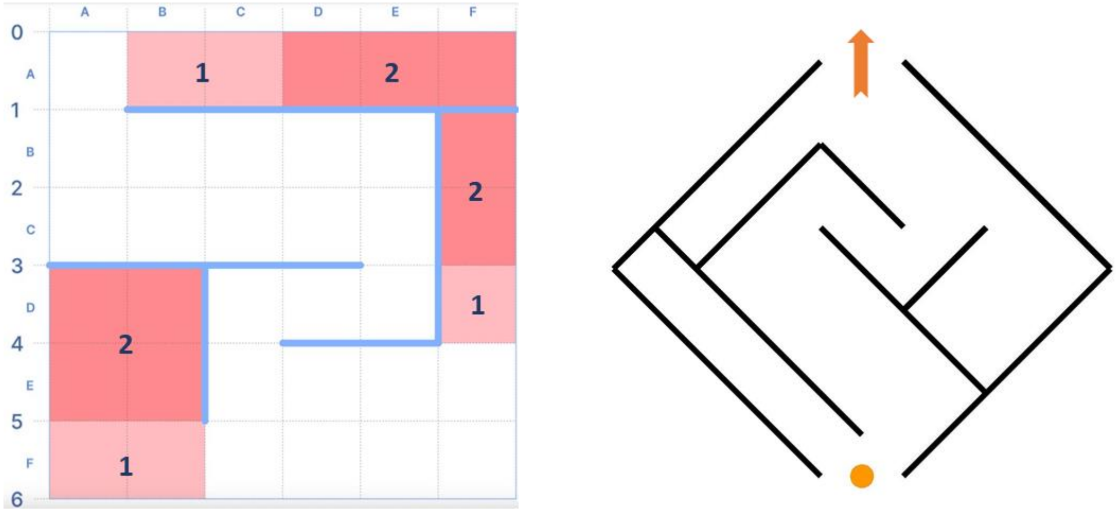

Figures S1. Maze setup and error calculation example. The numbers (1 or 2) correspond to errors rate and are based on deviance from the correct path. An error point is added every time a participant enters error zone 1 or turns around towards the entrance instead of walking towards the exit, and 2 points for error zone 2 [2]. Time measurement began when participants entered the maze and concluded when they exited. The maze trial score is calculated as 100-Time/5-Error, where, Time is limited to 3 minutes, and Error is the total error score during the trial. The participant can advance to the next maze only when Trial_Score ≥ 70.

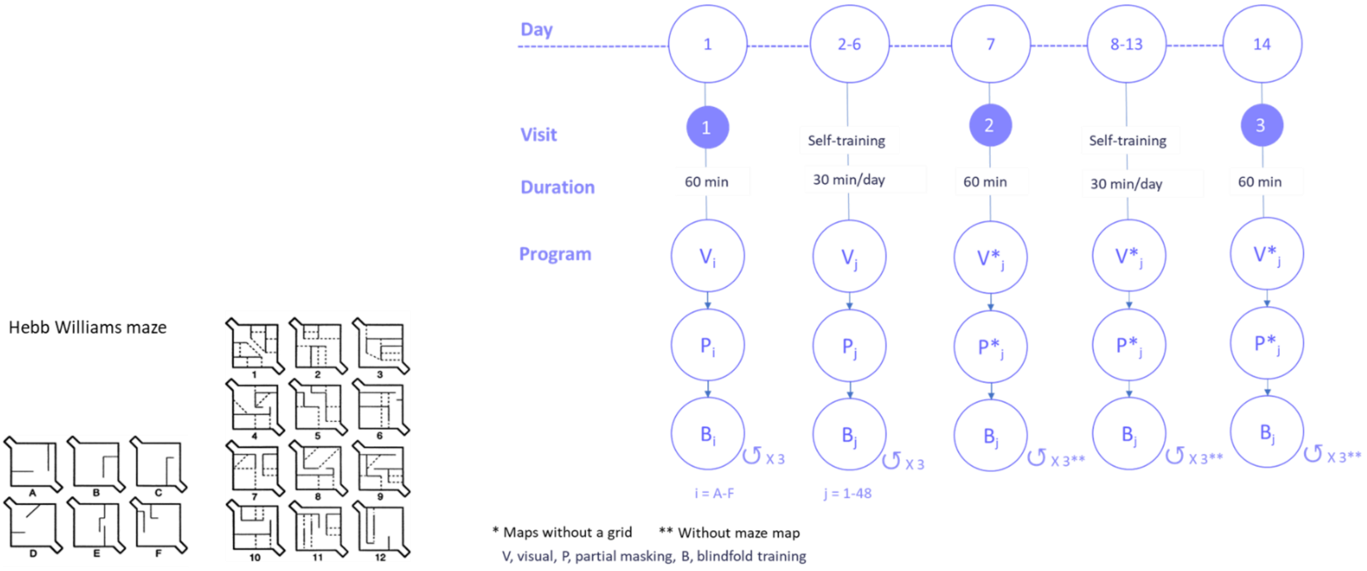

Figure S2. Maze training daily protocol scheme.

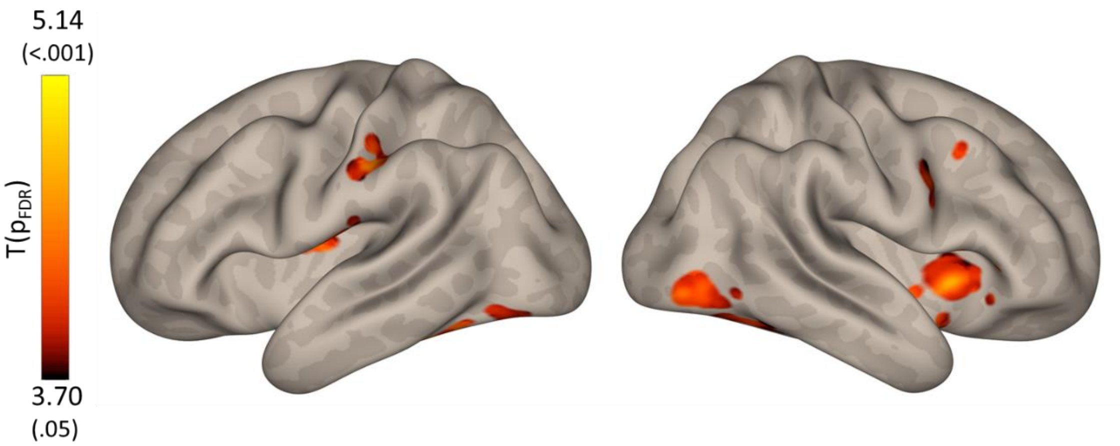

Fig S3. Seed-to-voxel R hippocampus connectivity maps of longitudinal differences. Seed: right hippocampus. POST > PRE-intervention of the resting state brain imaging data – group level. n = 17, $p < 0.05$, FDR corrected, parametric stats, two sided. See also Supplementary Table S2 for details.

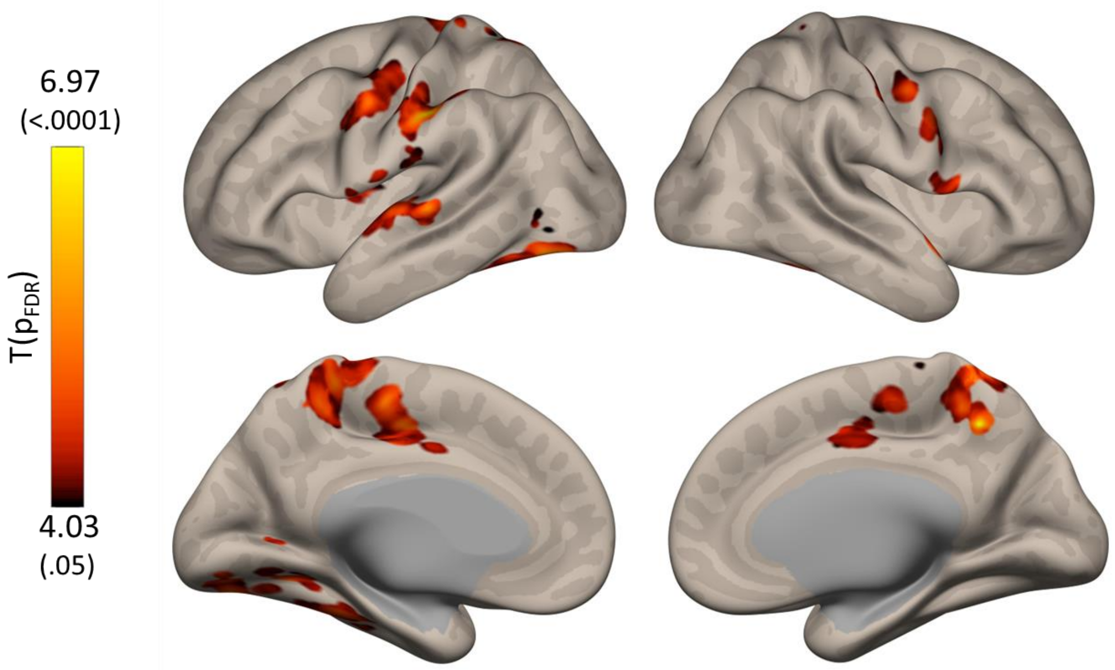

Fig S4. Seed-to-voxel L hippocampus connectivity maps of longitudinal differences. Seed: left hippocampus. POST > PRE-intervention of the resting state brain imaging data – group level. n = 17, $p < 0.05$, FDR corrected, parametric stats, two sided. See also Supplementary Table S2 for details.

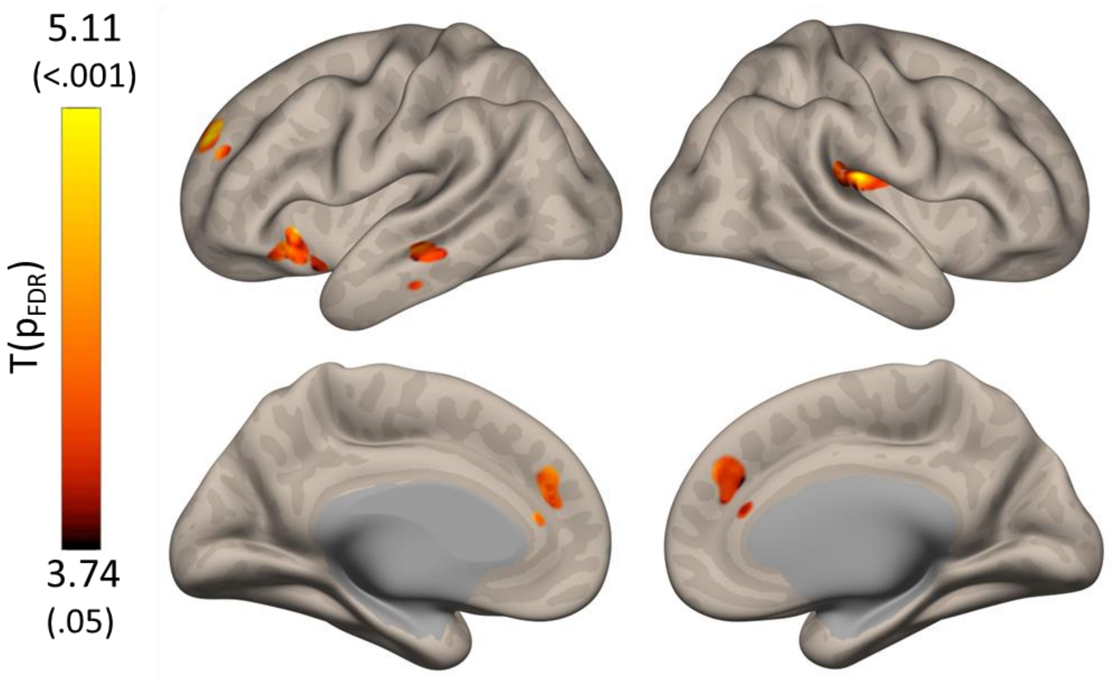

Fig S5. Seed-to-voxel R parahippocampus connectivity maps of longitudinal differences Seed: right parahippocampus Area3. POST > PRE-intervention of the resting state brain imaging data – group level. n = 17, $p < 0.05$, FDR corrected, parametric stats, two sided. See also Supplementary Table S2 for details.

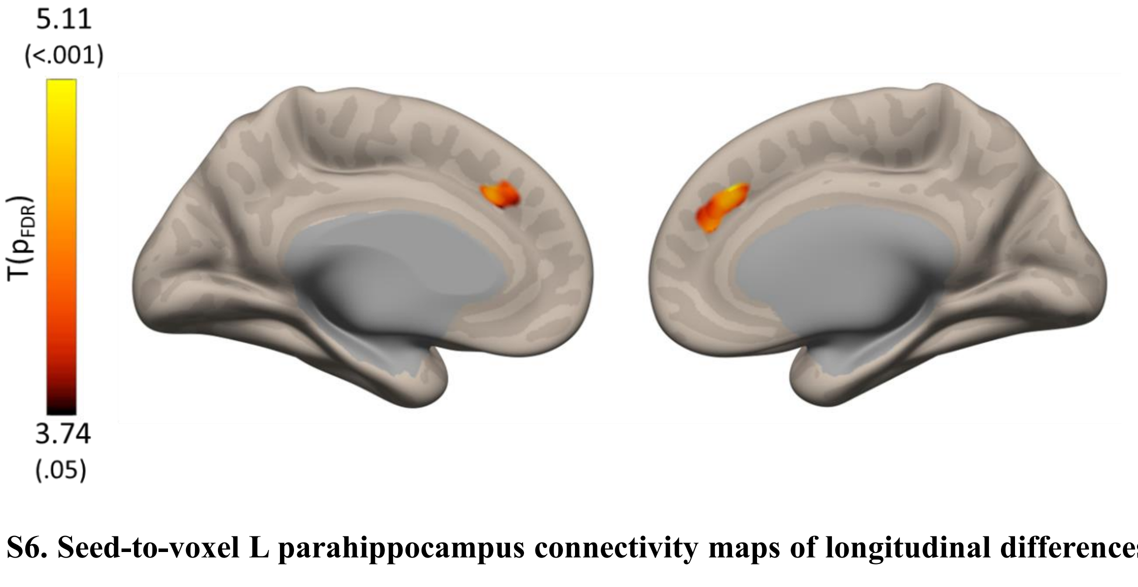

Fig S6. Seed-to-voxel L parahippocampus connectivity maps of longitudinal differences. Seed: left parahippocampus Area3. POST > PRE-intervention of the resting state brain imaging data – group level. n = 17, $p < 0.05$, FDR corrected, parametric stats, two sided. See also Supplementary Table S2 for details.

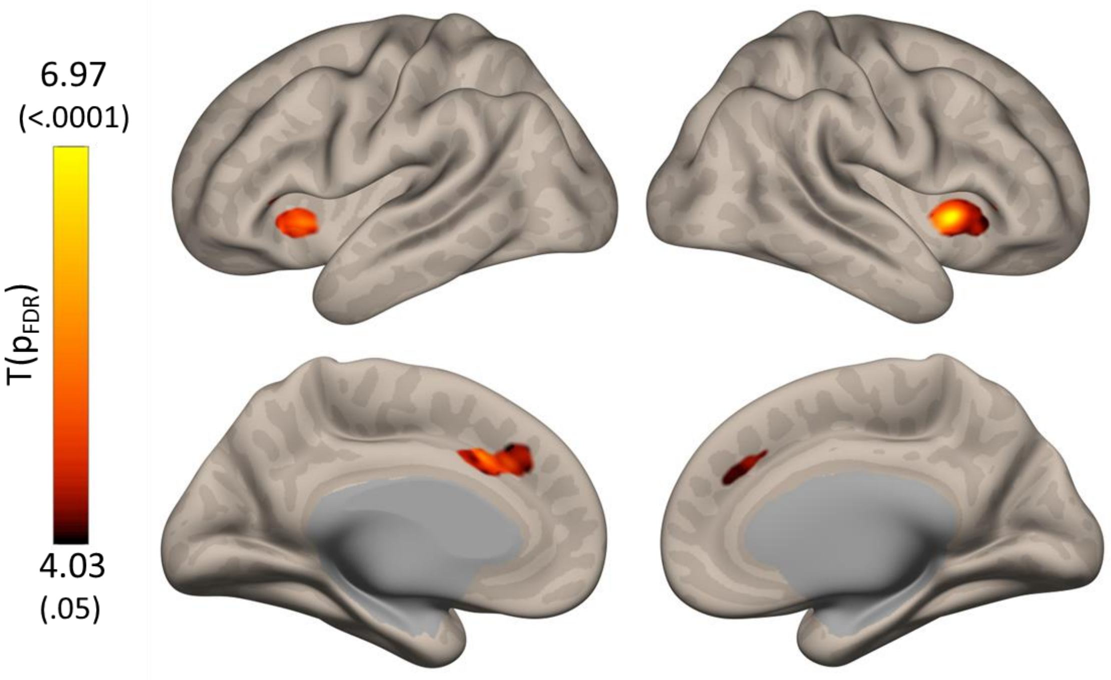

Fig S7. Seed-to-voxel R parahippocampus connectivity maps of longitudinal differences. Seed: right parahippocampal Area2. POST > PRE-intervention of the resting state brain imaging data – group level. n = 17, $p < 0.05$, FDR corrected, parametric stats, two sided. See also Supplementary Table S2 for details.

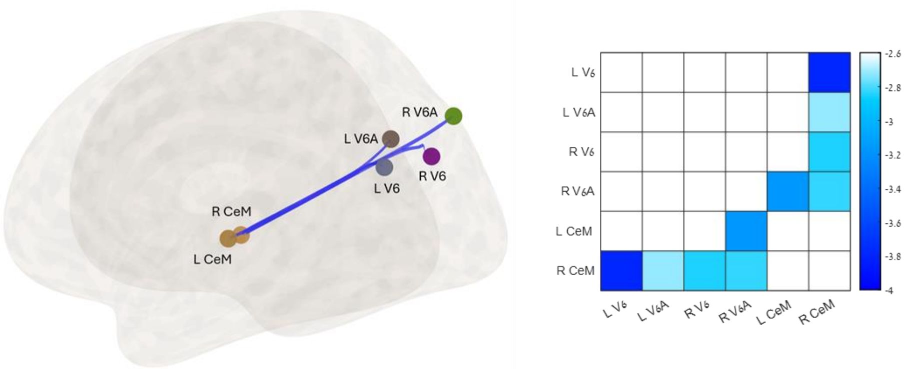

Fig S8. ROI-to-ROI navigation network analysis of longitudinal post-intervention differences. Brain network representation and a connectivity matrix of significant post-intervention alternations. A significant increased anticorrelation rsFC was demonstrated between the left and right visual area V6 and V6A and the central medial nucleus of the thalamus (CeM). CeM, central medial nucleus of the thalamus, POST > PRE-Intervention, n = 17, $p_{FDR} < 0.05$, (F NEG < -2.73), White = NS

3 References

1. Huang, C.-C., Rolls, E.T., Feng, J., and Lin, C.-P. (2022). An extended Human Connectome Project multimodal parcellation atlas of the human cortex and subcortical areas. Brain Structure Function *227*, 763-778.
2. Chebat, D.-R., Maidenbaum, S., and Amedi, A. (2015). Navigation using sensory substitution in real and virtual mazes. PloS one *10*, e0126307.
